# The Endemic Insular and Peninsular Species *Chaetodipus spinatus* (Mammalia, Heteromyidae) Breaks Patterns for Baja California

**DOI:** 10.1371/journal.pone.0116146

**Published:** 2014-12-26

**Authors:** Sergio Ticul Álvarez-Castañeda, Robert W. Murphy

**Affiliations:** 1 Centro de Investigaciones Biológicas del Noroeste, Instituto Politécnico Nacional 195, La Paz, Baja California Sur, México; 2 Centre for Biodiversity and Conservation Biology, Royal Ontario Museum, Toronto, Canada; University of Arkansas, United States of America

## Abstract

The Baja California peninsula is the second longest, most geographically isolated peninsula on Earth. Its physiography and the presence of many surrounding islands has facilitated studies of the underlying patterns and drivers of genetic structuring for a wide spectrum of organisms. *Chaetodipus spinatus* is endemic to the region and occurs on 12 associated islands, including 10 in the Gulf of California and two in the Pacific Ocean. This distribution makes it a model species for evaluating natural historical barriers. We test hypotheses associated with the relationship between the range of the species, patterns in other species, and its relationship to Pleistocene-Holocene climatic changes. We analyzed sequence data from mtDNA genes encoding cytochrome *b* (*Cytb*) and cytochrome *c* oxidase subunits I (*COI*) and III (*COIII*) in 26 populations including all 12 islands. The matrilineal genealogy, statistical parsimony network and Bayesian skyline plot indicated an origin of *C. spinatus* in the southern part of the peninsula. Our analyses detected several differences from the common pattern of peninsular animals: no mid-peninsula break exists, Isla Carmen hosts the most divergent population, the population on an ancient southern Midriff island does not differ from peninsular populations, and a mtDNA peninsular discordance occurs near Loreto.

## Introduction

The geography of the peninsula of Baja California (BCP) and surrounding islands has facilitated studies of the underlying patterns and drivers of genetic structuring of a wide spectrum of organisms. The peninsula, which stretches for more than 1,000 kilometers from southern California to Los Cabos, is the second longest and most geographically isolated peninsula on Earth [1], [2]. A mixture of both climatological and geological events appears to have strongly affected the associated biota [3], [4], [5]. For example, the peninsula may have been an insular archipelago [6] formed by the ephemeral midpeninsular Vizcaino Seaway and another marine inundation at the Isthmus of La Paz [7], [5]. Populations of many taxa also occur on adjacent islands in the Gulf of California and Pacific Ocean. These populations often differ phenotypically from their peninsular and mainland relatives and often exhibit some degree of genetic structure. Some insular populations have received taxonomic recognition as species or subspecies [8], [9].

Pleistocene–Holocene climatic cycling has influenced the geographic distributions and genetic structure of many species [10], [11]. During cooler and wetter glacial periods, xerophilic species could have had their ranges reduced and in warm interglacial conditions, and expanded, such as after the last glacial maximum (LGM; 21,000 years ago [12]). This has been termed the expansion-contraction model [13]. An understanding of how species previously responded to climate changes allows for the predicton of responses to future climate changes. Such knowledge will facilitate the development of conservation strategies. In the case of xerophilic species, this may involve the management of expanding ranges expected to occur in the near future. Mesophilic taxa are predicted to have contracting distributions [14], [15].

*Chaetodipus spinatus*, the spiny pocket mouse, is a heteromyid species that occurs widely on the BCP. The species also occurs on 10 islands in the Gulf of California and on two in the Pacific Ocean [8], [9], [16]. Xerophilic *C. spinatus*, the most common mouse on the BCP, mainly occurs in areas with stony soils at elevations from sea level up to 1,500 m [16], [17]. It is the only native mammal on some islands in the Gulf of California [18]. Populations of this species exhibit significant phenotypic variation, especially those forms occurring on islands, as evidenced by the existence of 18 subspecies [9], [16], [19], [20], [21], [22]. Its distribution makes it a model species for evaluating how natural historical barriers and/or geographic distance have played important roles in determining its genetic structuring. Here, we evaluate sequence data from the protein-coding mtDNA genes cytochrome *b* (*Cytb*) and cytochrome *c* oxidase subunits I (*COI*) and III (*COIII*) in 26 populations, including all insular occurrences. To facilitate comparisons with most other studies, we use analyses of mtDNA data only to assess the matrilineal history of this species. This approach tracks female dispersion and nuclear gene flow does not obscure this history [23]. We testet hypotheses concerningthe relationship between the range of the species in relation to the Pleistocene–Holocene climatic change and matrilineal history. Further, we investigated the spatial genetic structuring among peninsular and insular populations.

## Materials and Methods

### Sample collection

**Figure 1:**
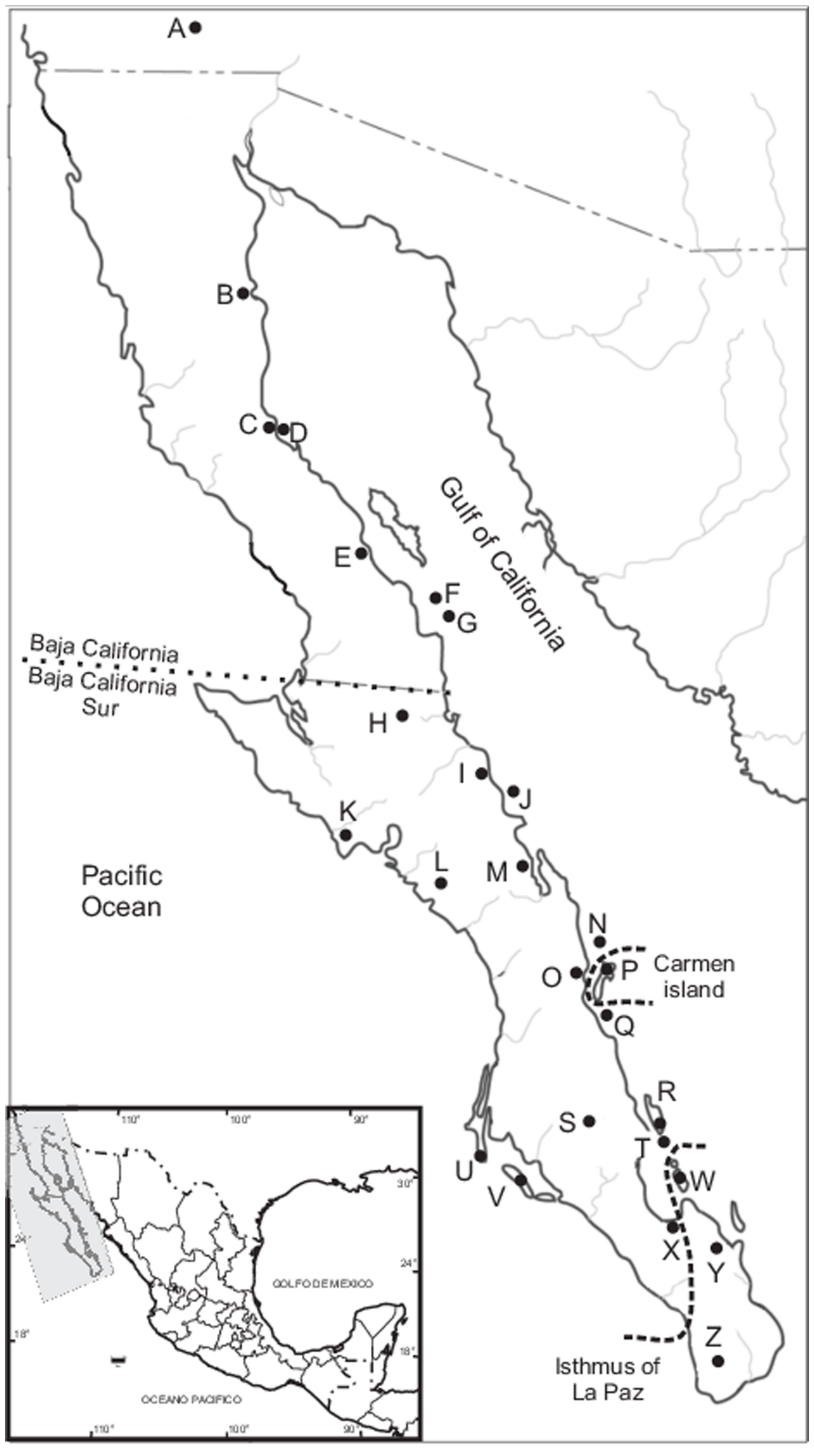
Map of the location of each of the 26 sampled localities of the *Chaetodipus spinatus*. United States: California: Tumco (A). Mexico: Baja California: San Felipe (B), Gonzaga Bay (C), Gonzaga Island (D), Bahía de los Angeles (E), Las Animas Island (F), San Lorenzo Island (G). Baja California Sur: Vizcaino (H), Santa Rosalia (I), San Marcos Island (J), Punta Abreojos (K), San Juanico (L), Mulege (M), Coronados Island (N), Loreto (O), Isla Carmen (P), Danzante Island (Q), San José Island (R), San Pedro (S), San Francisco Island (T), Magdalena Island (U), Margarita Island (V), Espiritu Santo Island (W), La Paz (X), Los Planes (Y), and Cape Region (Z).

Our analyses included 212 ingroup sequences obtained from 26 peninsular and insular populations (Fig. 1). These populations represented most of the geographic range of *C. spinatus* on the BCP and the surrounding islands. Specimens were collected using transects of 40 Sherman live traps. A maximum of five individuals of each population were collected and all others were released in the same place after capture because many insular populations are protected by Federal laws [24]. Voucher specimens were deposited in the Centro de Investigaciones Biologicas del Noroeste (CIB). In all instances, the handling and sacrifice of animals were performed according to the recommendations of the American Society of Mammalogists [25]. The collecting of specimens and method of euthanasia (cervical dislocation [53]) was conducted under the permit FAUT-044 from the Secretaria del Medio Ambiente y Recursos Naturales, México (SEMARNAT). Permit SGPA/DGVS/04192/13 allowed work with the threatened population.

### Laboratory procedures

Genomic DNA was extracted from muscle tissue that was maintained at −20°C in 70% ethanol using DNeasy kits (QIAGEN Inc., Valencia, CA) following the manufacturer's protocols. The first ∼800 bp of *Cytb* was amplified with primer pairs MVZ05/MVZ16 and the second ∼600 bp with MVZ127/MVZ14 [26], [27]. The concatenation of both fragments resulted in the complete 1140 bp of *Cytb*. A 700 bp fragment of *COIII* was amplified with primer pair L8618/H9323 [28] and a fragment of *COI* (657 bp) with primers LCO1490/HCO2198 [29].

The following conditions for initial double-stranded amplifications were used: 12.5 µl (10 ng) template, 4.4 µl ddH_2_O, 2.5 µl each primer (10 nM), 0.474 µl (0.4 nM) dNTPs, 0.5 µl (3 mM) MgCl_2_, 0.125 µl Taq polymerase, and 1x Taq buffer to a final volume of 25 µl. Amplification conditions consisted of 3 min initial denaturation at 94°C followed by 37 cycles of denaturation at 94°C for 45 sec, 1 min annealing at 50°C, and 1 min extension at 72°C. Double-strand DNA was cleaned using a QIAquick PCR Purification Kit (QIAGEN Inc., Valencia, CA).

The templates were cycle-sequenced with MVZ05/MVZ16 and MVZ127/MVZ14 for *Cytb* amplifications, L8618/H9323 for *COIII* and LCO1490/HCO2198 for *COI*. Reverse strands MVZ16, MVZ14, H9323 and HCO2198 were sequenced to confirm accuracy. All sequencing used a Big Dye Terminator Kit and was run on an ABI 3730 automated sequencer following the manufacturer's protocols.

### Alignment and haplotype determination

Nucleotide sequences were aligned using sequencher v.3.1 (Gene Codes Corp., Ann Arbor, Michigan), visually inspected, and translated to amino acids for alignment confirmation. Missing data were coded with a question mark. Nonredundant haplotypes were identified with collapse v.1.1 [30] (http://darwin.uvigo.es).

Levels of genetic variation within subspecies of *C. spinatus* were measured in terms of number of polymorphic sites, nucleotide diversity (Φ per nucleotide site, i.e., the probability that two randomly chosen homologous nucleotides differed [31]), haplotype diversity (h), and number of private haplotypes using arlequin v.2.0 [32]. Non-redundant haplotypes were deposited in GenBank under accession numbers KM980203–KM980438.

### Inter- and intraspecific variation

Two separate analyses were conducted. First, a phylogenetic analysis was performed for concatenated (2157 bp) fragments of *Cytb* (800 bp), *COIII* (700 bp) and *COI* (657 bp). The analyses included 25 haplotypes. Second, a statistical parsimony network was constructed for the 800 bp fragment of *Cytb* from 212 specimens, the 700 bp fragment of *COIII* from 46 specimens, and the 657 bp fragment of *COI* from 112 specimens. The networks were built using the most proximal geographic link.

Phylogenetic reconstructions based on maximum likelihood (ML) and Bayesian inference (BI) were performed with non-redundant haplotypes for each population. ML trees were constructed using paup* v.4.0b10 [33]. BI trees were constructed using MrBayes v.3.0b4 [34]. Separate analyses were conducted for each gene and the concatenated data. Substitution models were estimated with the Akaike information criterion (AIC) as implemented in MrAIC [35]. Metropolis-coupled Markov chain Monte Carlo (MCMC) sampling was performed with four chains run for 5 million iterations using default model parameters as starting values. We sampled trees every 1,000th iteration and discarded the first 1,000 trees as burnin after the chains reached stationarity. Bayesian posterior probabilities, the frequency of nodal resolution, were taken from the 50% majority rule consensus of sampled trees. ML was performed using a heuristic search with 1,000 replicates and swapping with the TBR algorithm. Reliability was assessed using each of the three codon positions separately while applying equal weights. Nodal support was assessed using nonparametric bootstrapping. Trees were rooted using *Chaetodipus arenarius, C. siccus, C. dalquesti, C. fallax* and *C. californicus*, which were found to be sister groups of *C. spinatus* [36].

The relationships among *Cytb* haplotypes were inferred using statistical parsimony [37] implemented in tcs v.2.8 [38]. Tcs estimated haplotypic relationships given low levels of divergence and provided a *P* = 0.95 plausible explanation for all connections. The null distribution to test for the significance of the variance components and pairwise *F*-statistic equivalents (*FST*) were constructed from 10,000 permutations.

Tajima's *D* [39] and Fu's *Fs* [40] tests were performed in arlequin v.3.5 to detect departures from neutrality or from a Wright-Fisher population model. Departure from neutrality may have been caused by hitchhiking, population size expansion, background selection or a selective sweep [41]. Negative and significant values of Fu's *Fs* and Tajima's *D* were expected from samples that had undergone recent demographic expansions or showed certain departures from neutrality [41]. These tests were taken together with plots of pairwise differences and mismatch distributions [42] as evidence of demographic change and/or deviations from neutrality.

A coalescent-based approach using Bayesian skyline plots was performed to estimate the posterior distribution for effective population sizes of *C. spinatus*, thus allowing inferences of population fluctuations over time throughout the peninsula. This analysis was performed for all *Cytb* haplotypes of *C. spinatus* (*n* = 133). Program settings were created using BEAUti v.1.7.4 within beast [43]. The MCMC analysis was run with the following settings: GTR substitution model, estimated base frequencies, gamma site heterogeneity model with four categories, a Bayesian skyline coalescent tree prior with 10 groups, and piecewise-constant skyline model. We estimated a) standard deviation of 0.178 (95% highest posterior density interval: 0.000–0.458) for the uncorrelated lognormal relaxed clock. The strict molecular clock model was used in the MCMC analysis. The substitution model (GTR + gamma) was selected using MrModeltest v.2 [35]. A divergence rate of 1.3 mutations per 1.0×10^5^ years was used [44] per lineage per site per year. Chains were run for 10^8^ generations while sampling the parameter every 10^3^ generations and the first 10% was discarded as the burnin. The MCMC analysis was repeated four times using different random number seeds to determine if the independent runs converged on the same distribution. The four independent runs were combined using LogCombiner v.1.7.4 (in beast). The Bayesian skyline plot was created using tracer v.1.5 (http://tree.bio.ed.ac.uk/software/tracer).

## Results

### Phylogenetic relationships

**Figure 2:**
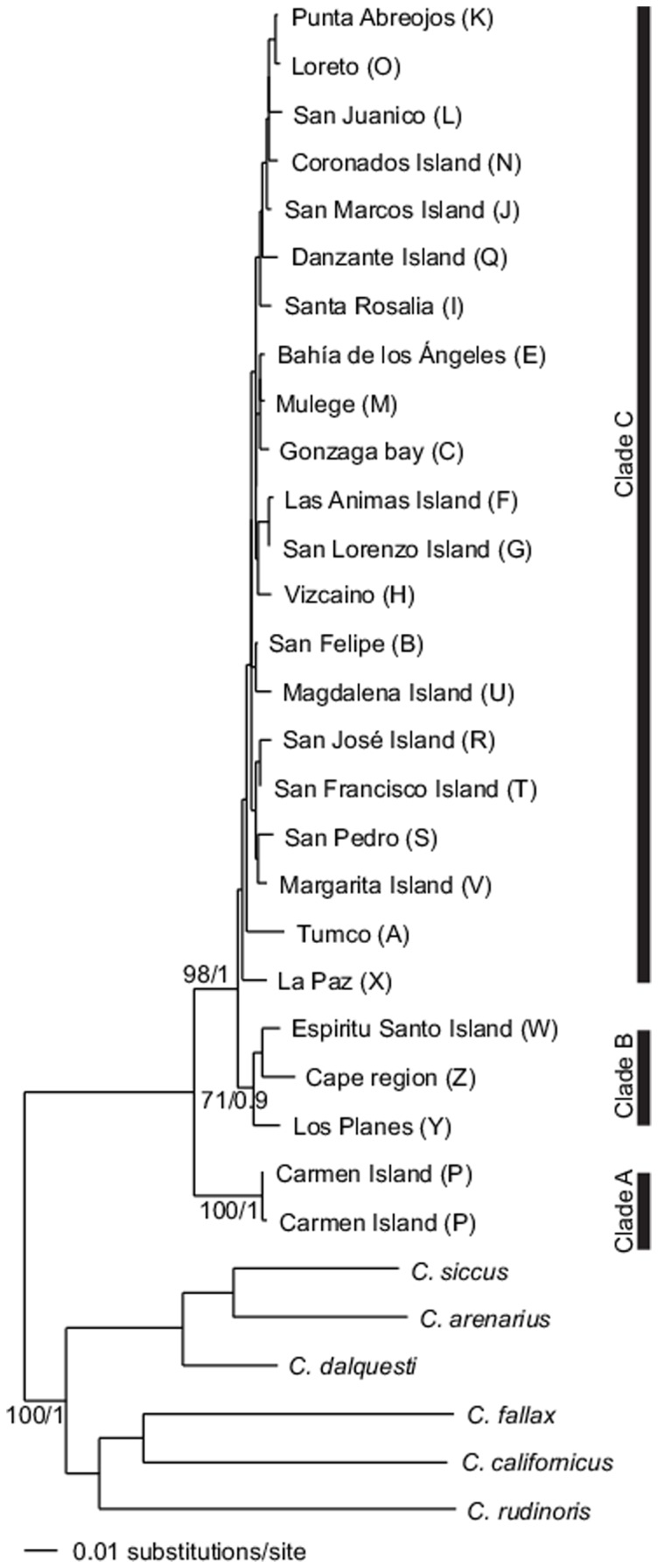
Phylogram of 800 bp of the cytochrome *b* gene shows three main lineages that are the most constant in all analyses. Note the monophyly of all *Chaetodipus spinatus* (Maximum-likelihood/Bayesian inference/Bootstrap). Lineage A involves Isla Carmen only, lineage B occurs south of the Isthmus of La Paz and lineage C comprises all other population, including two haplotypes from Isla Carmen. Locality names and letters at the tip of each branch are shown in Fig. 1.

The model of sequence evolution that best fit our sequence data was GTR + gamma for the three genes and the concatenated data. BI and ML trees for *Cytb*, *COIII*, *COI* and the concatenated data converged on an essentially identical topology. Three lineages were resolved (Fig. 2): Lineage A had 2 haplotypes only, both from Isla Carmen; Lineage B contained specimens from the Cape Region, the area south of the Isthmus of La Paz; and Lineage C was comprised of all other haplotypes from all other areas of BCP and all other islands.

### Matrilineal History

#### Genetic variation

*Cytb* consisted of 800 bp fragments for 212 specimens, among which 103 (12.87%) were polymorphic. The average transition–transversion ratio was 3.06, and nucleotide frequencies were A = 26.44%, C = 26.45%, G = 14.15%, and T = 32.96%. The data for *C. spinatus* resolved 133 unique haplotypes among the 212 specimens representing all 26 localities. Pairwise differences averaged 12.76±5.77 and π averaged 0.015±0.007.

**Figure 3:**
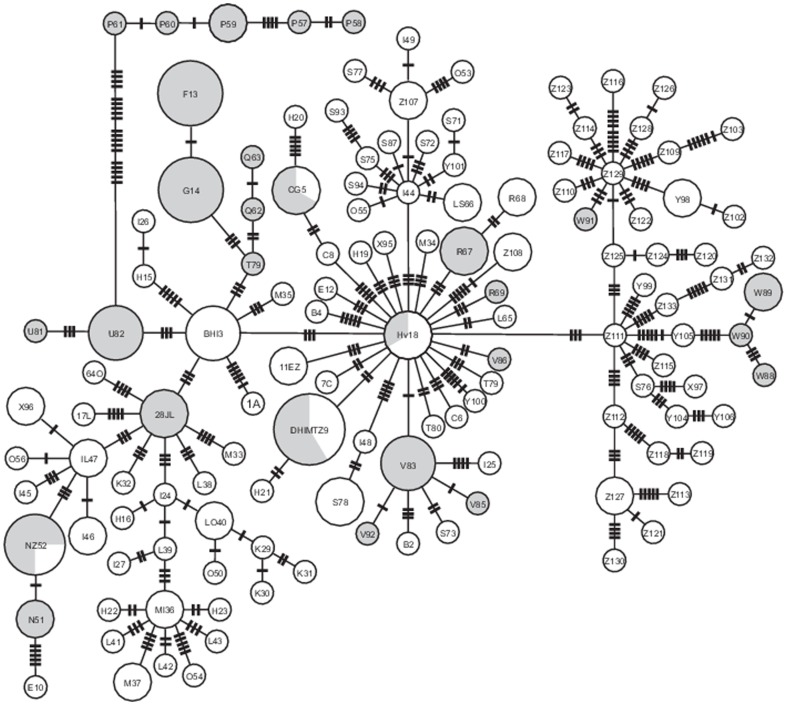
A statistical parsimony network of 133 unique haplotypes recovered from the 800 bp of *Cytb* sequence data that include all sampled populations of *Chaetodipus spinatus* from the Baja California peninsula and surrounding islands. Transverse lines on branches indicate the number of base substitutions. The percentage of gray color per haplotype indicates the number of specimens found in the islands and the white haplotypes are from the peninsula area. Each haplotype is denoted by a number (see Appendix 1) and letter (from Fig. 1).

The statistical parsimony network connected 133 unique haplotypes through a maximum of 19 mutational steps (Fig. 3). The network did not depict clear genetic structure in relation to subspecies or geography, except for Isla Carmen, which separated from all other populations of *C. spinatus* by 19 steps (Fig. 3). Most haplotypes (105, 79.5%) occurred in one site only. Twenty seven haplotypes (20.4%) occurred in more than one locality. Haplotype 9 was the most widely distributed lineage, occurring in 12 peninsular sites from Bahía San Luis Gonzaga (locality C) southwards to Los Barriles (Z), including the islands of San Luis Gonzaga (D), San Marcos (J), San Francisco (Q) and Margarita (V, Fig. 1). Insular and peninsular specimens shared three haplotypes: haplotype 5 was common to Bahia Gonzaga (C) and Las Animas Island (also known as San Lorenzo Norte) (G); haplotype 18 occurred at San Francisco de la Sierra (H) and on Margarita Island (V); and haplotype 52 was found at Cabo (Z) and Coronados Island (N). More widely distributed haplotypes occurred on the northern peninsula and this area contained few haplotypes. Greater diversity occurred in southern areas where haplotypes exhibited narrow distributions.

The network depicted aggregated clusters of haplotypes throughout the peninsula with one central haplotype and many peripheral ones separated by few mutational steps. This indicated a continuing spreading of the species throughout the range. In three clusters, the central haplotype was endemic to an island and peripheral haplotypes occurred on other islands and the peninsula. Only one cluster was restricted to one area: the haplotypes of Isla Carmen. The right side of the statistical parsimony network contained all haplotypes that had at least one specimen from the Cape Region. Among islands, only Isla Carmen contained a single lineage of haplotypes, of which there were five.

**Figure 4:**
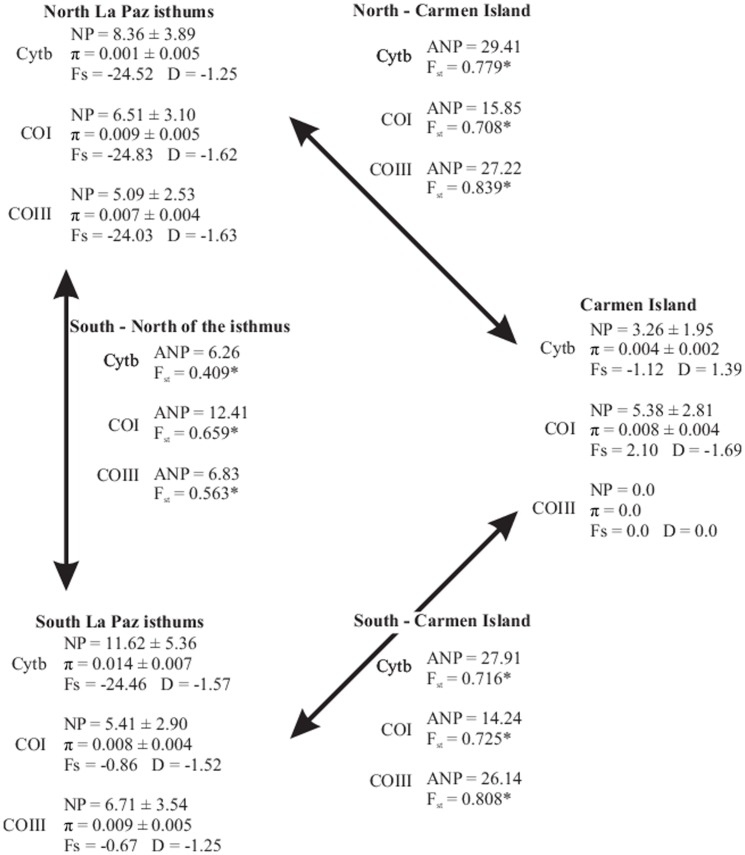
Genetic parameters within and among the three main geographical areas for *Cytb*, *COI* and *COIII* including the mean number of pairwise differences (NP), nucleotide diversity (π), Tajima's *D*, Fu's *Fs*, average number of pairwise differences between populations (ANP) and the fixation index (F_ST_). *Significance level  = 0.05.

The statistical parsimony network for *COI* involved 112 specimens, 59 haplotypes, a maximum of 49 mutational steps, pairwise differences of 11.17±4.37, and a nucleotide diversity averaging 0.017±0.008. For *COIII*, the network involved 46 specimens, 37 haplotypes, 46 maximal steps, pairwise differences averaging 9.35±5.11 and nucleotide diversity of 0.013±0.006. The patterns in both networks were similar patterns to those for *Cytb* (Fig. 4). Again, the population of Isla Carmen separated from other populations by 27 steps for COIII.

The Cape Region was the most haplotype-rich area and most variation occurred in the lowlands. Variation occurred throughout the peninsula, but was concentrated in the south. This pattern suggested the southern part of the BCP may have been an interglacial refugium during the last glacial maximum.

#### Neutrality tests

**Figure 5:**
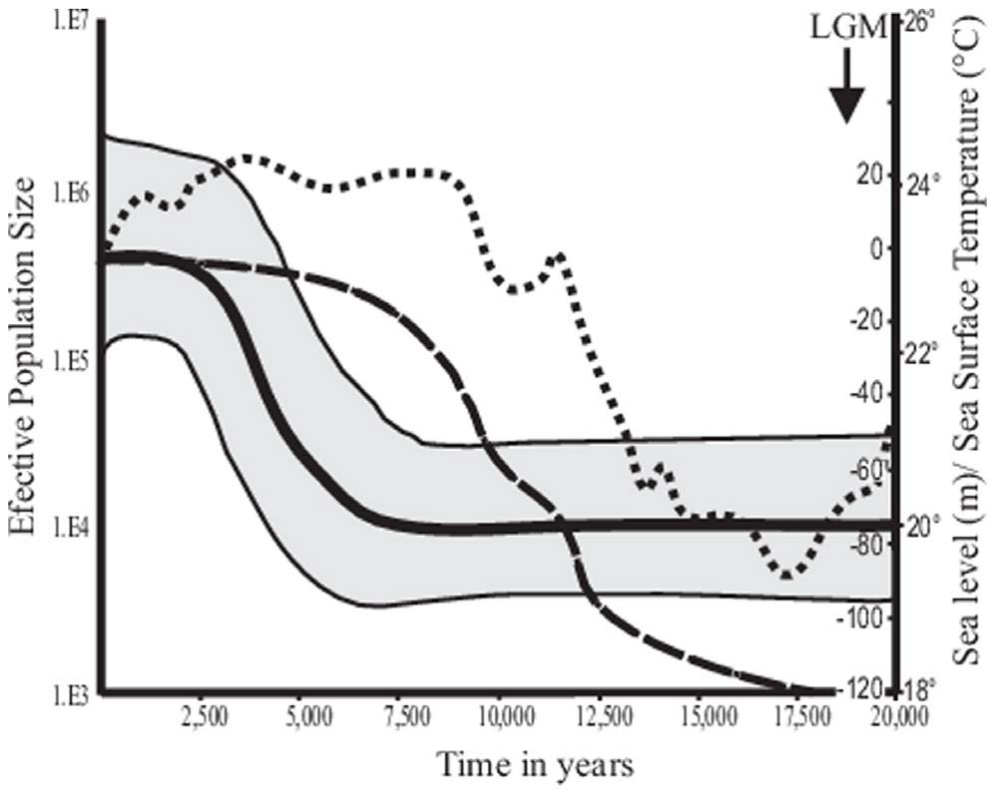
Bayesian skyline plot for *Chaetodipus spinatus* from the 133 haplotypes showing the median value of the log10 of the effective population size as function of time. Shaded area is the 95% highest posterior density. The dashed line represents change in sea-level (Fairbanks 1989) and the dotted line shows the change in the sea-surface temperature (Hebert et al. 2001).

Tajima's *D* and Fu's *Fs* for the three genes suggested that populations experienced strong demographic expansions (Fig. 4). Expansion values were not the same along the entire peninsula because greater values occurred north of the Cape Region. The Bayesian skyline plot suggested an increased effective population size many years after the LGM at about ∼7.500 Ka. Population expansions followed a period of effective stability (Fig. 5). This discovery corresponded with values of Tajima's *D* and Fu's *Fs* and the patterns in the statistical parsimony network of some central haplotypes in that many peripheral haplotypes were separated from central ones by few mutations steps.

## Discussion

Understanding the complex geology of the BCP and associated islands remains an ongoing problem. For example, early estimates for the origin of the Angel de la Guarda block in the Gulf of California were revised from being approximately 1 Ma [7], [45] to 8–5 Ma [46], [47], [48]. Further, much of the older stratigraphic history remains elusive after being overwritten by more modern geologies, such as Holocene volcanism and other forms of orogenesis. Because of this, the genetic patterns of the biota may help infer the historical stratigraphy of the peninsula. Together, inferences from geology, climate and biological patterns may lead to the identification of common patterns and the formation of testable hypotheses for the drivers of organismal variation.

The dominant genetic pattern on the BCP consists of a midpeninsular mtDNA discordance, and another one at the Isthmus of La Paz [5], [49]. These discordances occur in most species of animals [49], [50] and taxa not exhibiting this discordance usually show a pattern of recent northwards dispersal [5]. Whereas stratigraphic data and magnetic anomalies converge on a date for the Cape Region event(s), genetic patterns have been used to infer the midpeninsular discordance and the possible, ephemeral Vizcaino Seaway [5], [7]. Two additional discordances exist in some taxa: near Loreto and in the southern Cape Region [51].

Vicariance events at the Isthmus of La Paz due to marine incursions date to around 15 Ma [6]. Unlike this event, much controversy surrounds the dating of the midpeninsular discordance. The initial date of about 1 Ma by Upton and Murphy [7] was based on a calibration for the formation of the Angel de la Guarda block. Discarding such dates (e.g., [52], [53]), current estimates place the midpeninsular discordance to 7.1±0.05 Ma [53] or from 9.0 to 4.5 Ma [54]. This variation may reflect one or two events, assuming that all taxa evolve at the same rate [55]. The other two genetic discordances were more recent than the midpeninsular disruption [52], [56]. All analyses show greater genetic divergence at the Isthmus of La Paz than the middle peninsular area for most mammals [4], [5], [57], [14].

For landbridge islands, their history is unambiguous. Sea level changes associated with Pleistocene glacial–interglacial climatic cycles formed and broke these insular connections with the mainland, but not all [58], [59]. At times of maximum glaciation, landbridges connected many islands to the adjacent mainland about 18 Ka when the sea level was 120 m lower than now [60]. During this time, biotic interchange is likely to have occurred [5], [18]. The higher interglacial sea levels of today have been more or less constant since 5 Ka (Fig. 5); landbridge insular populations have been isolated for at least that amount of time.

The origin and history of older, deep-water islands is more complex. Biological dating of these islands remains difficult because of over-sea dispersal of species and anthropogenic translocations [5], [52]. The occurrence of *C. spinatus* on the southern Midriff, San Lorenzo archipielago, breaks the normal pattern of substantial genetic differentiation seen in most other taxa. For example, most squamate reptiles on the island differ notably from their peninsular counterparts [5]. One exception to this pattern is the absence of genetic differences between the chuckwalla, *Sauramalus hispidus*, on the San Lorenzo and Salsipuedes islands versus the species on Angel de la Guarda Island. This has been attributed to the translocation of chuckwallas from the later island to the former by the Seri people thousands of years ago [5]. Translocations of large edible lizards was not unusual for the Seri [61], [62].

### History

Tajima's *D* and Fu's *Fs* provide strong signals of population expansion on the peninsula for *C. spinatus* as do the Bayesian skyline plots. As expected, insular haplotypes do not have relationships among themselves but rather they associate with nearby parts of the peninsula. Further, insular populations have fewer haplotypes than areas on the adjacent mainland, possibly due to bottlenecking during the last 12–5 Ka, when the sea level increased and began the isolation process. The absence of insular differentiation, except for Isla Carmen, also points to recent isolation.

The high expansion values found on the peninsula are in contrast to the low numbers of haplotypes on the islands. Most islands with *C. spinatus* are located near the peninsula and the areas share similar habitats. Thus, insular populations could exhibit greater diversity. However, limited immigration may affect overall diversity and low levels of variation on recently colonized deep-water islands, such as the southern Midriff islands. The low level of genetic diversity may reflect a founder effect. Islands larger than 100 km^2^ have a greater number of haplotypes than those of smaller areas (Table 1). In this case, inbreeding may be reducing variation in the small islands.

**Table 1:** Sample size (N), number of haplotypes (H), number of haplotypes exclusive to that population (E), mean number pairwise differences (NP), nucleotide diversity (π), number of polymorphic sites (P), number of observed sites with transitions (Tt) and number of observed sites with transversions (Tv).

Group	N	H	E	E/H	NP	π	P	Tt	Tv
A	1	1	1	1.0	0.00±0.00	0.000±0.000	0	0	0
B	3	3	2	0.7	15.00±9.31	0.018±0.0145	23	15	8
C	5	4	3	0.8	5.60±3.23	0.007±0.004	11	10	1
**D**	**5**	**1**	**0**	**0.0**	**0.00±0.00**	**0.000±0.000**	**0**	**0**	**0**
E	3	3	2	0.7	8.66±5.52	0.010±0.008	12	8	4
**F**	**9**	**1**	**1**	**1.0**	**0.00±0.00**	**0.000±0.000**	**0**	**0**	**0**
**G**	**9**	**2**	**1**	**0.5**	**1.11±0.79**	**0.001±0.001**	**5**	**5**	**0**
H	12	10	7	0.7	6.87±3.48	0.008±0.004	31	25	6
I	13	10	7	0.7	5.56±2.90	0.007±0.004	15	13	3
**J**	**4**	**1**	**1**	**1.0**	**0.00±0.00**	**0.000±0.000**	**0**	**0**	**0**
K	4	4	4	1.0	5.56**±**3.43	0.007**±**0.005	11	10	1
L	11	11	6	0.5	10.25**±**5.07	0.012**±**0.007	43	30	10
M	7	6	4	0.7	8.43**±**4.42	0.010**±**0.006	19	16	3
**N**	**5**	**2**	**1**	**0.5**	**0.60**±**0.56**	**0.001**±**0.001**	**1**	**1**	**0**
O	7	7	6	0.9	10.28**±**5.35	0.012**±**0.007	28	21	7
**P**	**6**	**5**	**5**	**1.0**	**3.26**±**1.95**	**0.004**±**0.002**	**6**	**4**	**2**
**Q**	**6**	**2**	**2**	**1.0**	**0.33**±**0.43**	**0.001**±**0.001**	**1**	**1**	**0**
**R**	**6**	**4**	**4**	**1.0**	**4.80**±**2.72**	**0.006**±**0.003**	**11**	**8**	**3**
S	13	11	10	0.9	8.32**±**4.12	0.010**±**0.005	39	31	9
**T**	**6**	**3**	**2**	**0.7**	**1.33**±**0.95**	**0.001**±**0.001**	**4**	**4**	**0**
**U**	**5**	**2**	**2**	**1.0**	**1.20**±**0.90**	**0.001**±**0.001**	**3**	**1**	**2**
**V**	**9**	**5**	**4**	**0.8**	**1.27±0.88**	**0.001±0.001**	**5**	**5**	**0**
**W**	**5**	**4**	**4**	**1.0**	**5.80±3.33**	**0.007±0.004**	**14**	**10**	**4**
X	6	5	5	1.0	11.40±6.03	0.014±0.008	28	23	5
Y	9	8	8	1.0	13.43±6.65	0.016±0.009	42	36	6
Z	34	29	26	0.9	10.43±4.85	0.012±0.006	57	47	12
All	203	133	103	0.8	12.75±5.77	0.015±0.007	103	87	29

Insular locations are in bold.

### Genetic variation

*Chaetodipus spinatus* exhibits a low level of genetic diversity for *Cytb* (π = 0.0125±0.006, *n* = 190) relative to other peninsular species of *Chaetodipus*, including *C. ammophilus* (π = 0.0164±0.008, *n* = 35), *C. arenarius* (π = 0.0185±0.009, *n* = 94), and *C. fallax* (π = 0.0228±0.011, *n* = 61). This is evident even though there are many more insular populations of *C. spinatus* than *Peromyscus* and *Dipodomys* spp. species (Alvarez-Castañeda, unpublished data), which could harbor historical variation.

Isla Carmen stands alone in its pattern of haplotypic diversity. All five matrilines on the island (57 to 61; see Fig. 3) come from the same common ancestor. Up to eight mutations separate them from one another indicating long-term isolation. Further, these matrilines differ from all other populations of *C. spinatus* by 19 mutations. Consequently, the population on Isla Carmen appears to have been isolated from the peninsula long ago. One founding linage may have diversified subsequently. Alternatively, the island may have once connected to the peninsula and after a long time of separation, the historical connections were erased. In either scenario, this occurrence is rare among the biota of the region, yet not unprecedented. Matrilineal patterns of orange-throated lizards associated with *Aspidoscelis hyperythra* show an origin near Isla Carmen followed by a relatively recent northward dispersal from the region [5], [63]. Whereas Soulé and Sloan [64], among others, speculated that Isla Carmen had a landbridge to the peninsula at times of glacial maxima, the biological patterns suggest an alternative scenario of long-term isolation. If a connection existed, it is unlikely to have been recent as the island and peninsula do not share haplotypes. The area may have a complex stratigraphic history. *Callisaurus draconoides* [54], *Urosaurus nigricaudus* [51], and *Phyllodactylus nocticolus* [56] have mtDNA discordances in the nearby peninsular region and other taxa may exhibit the pattern as well (Lorenzo pers. com.). Other taxa may also display patterns of long-term isolation on Isla Carmen along with mtDNA discontinuities in this area of the peninsula. These possibilities require testing.

The statistical parsimony network shows insular haplotypes associated with nearby peninsular ones. This pattern suggests that all colonization of the islands occurred from the peninsula, or that isolation occurred simultaneously for the affected islands due to increasing sea levels. In some cases, the close relationship among haplotypes (one or two mutational steps) occurs among nearby geographical areas; this implies dispersal among islands or between the mainland and islands. Some insular and peninsular haplotypes span a distance of 450 km, such as those from the islands of the San Lorenzo archipelago, (a.k.a. Islas Salsipuedes, Anima, San Lorenzo, and Roca Blanca [65]) and San Jose Island, and from San Marcos Island to Margarita Island. Direct dispersion among islands seems unlikely given geographic distances in the first case, and because the latter pair occurs on different sides of the peninsula. The sharing of these haplotypes suggests the occurrence of older, wide-spread matrilines.

Haplotype DHIMTZ9 occurs on many islands and in multiple peninsular populations (Fig. 3). Its wide-spread distribution implies recent gene flow, perhaps associated with the movement of *C. spinatus* from the southern part of the peninsula northwards. Values of Tajima's *D* (−1.22) and Fu's *Fs* test (−23.99) are congruent with this scenario. However, the mismatch distributions do not detect significant differences between the observed and model frequencies (α = 0.05; 100 replicates).

The Bayesian skyline plot indicates an increase in the effective population size about 7.5 Ka (Fig. 5). Consequently, the isolation of some islands from the peninsula might be more recent than previously thought. In congruence, Bayesian skyline plots suggest that some islands having *C. spinatus* became isolated ∼5 Ka. Other species of mammals with similar haplotypic distributions include *Ammospermophilus leucurus* for San Marcos Island [66], *Dipodomys merriami* from Margarita and San Jose islands [67], *Lepus insularis* on Espiritu Santo (Lorenzo pers. com.) and *Lepus californicus* from Cerralvo Island [68]. Notwithstanding, many insular haplotypes of rodent species do not occur on the mainland but rather they are endemic to specific islands [18], [66], [67]. Thus, all evidence suggests that insular populations of *C. spinatus* have been long-isolated from the peninsula. The occurrence of peripheral haplotypes on the network supports this scenario (Fig. 3).

### Biogeographic implications

Our analyses have biogeographic implications that extend beyond those for *C. spinatus*. Hafner and Riddle [69], [70] proposed that volcanic activity, possibly along with dramatic ecological and climatic changes [71], may explain coincidental midpeninsular mtDNA discordances. These scenarios require the simultaneous isolation of multiple species for an amount of time sufficient for genetic mutations to accumulate. Most populations on landbridge islands do not differ in terms of their mtDNA from peninsular populations after more than 5 Ka of isolation. Thus, more than 5 Ka of continual isolation is necessary to accumulate mutations via the exclusion of gene flow. Today, the proposed volcanic barrier—the Tres Virgenes volcanic field—contains many species of animals and plants despite substantial eruptions as recently as 30.7 Ka [72] (not 6515±75 yBP [73]) and subsequently in 1746 [74]. Further, extirpated species may rapidly recolonize areas of volcanic eruptions following the subsidence of biologically catastrophic volcanism. For example, San Benedicto Island in the Pacific Ocean southwest of BCP is only 10 km^2^ in size. It experienced a severe volcanian eruption with pyroclastic flows lasting from 1 August 1952 until 24 February 1953 [75]. Although the terrestrial biota of the island was completely wiped out, seabirds soon recolonized the island and plants either recolonized or merely reappeared, such as some endemic plants [75], [76], [77]. The Tres Virgenes volcanic field was not continually active for thousands of years and source biota likely remained nearby to recolonize the area rapidly. Volcanism would not isolate populations for a period of time sufficient to isolate populations and preclude gene flow, dispersion and dispersal. Thus, cataclysmic volcanism fails to explain the midpeninsular genetic discordance. Ecological and climatic changes may not explain the pervasive midpeninsular mtDNA discordance. This scenario requires significant functional selection on mtDNA and especially protein-coding genes involved in energy production, i.e. oxidative phosphorylation (OHPHOS). Both nuDNA and mtDNA genes involved in OXPHOS must co-evolve to preclude cytonuclear discordance and substantially inefficient energy production if this explanation has merit. Recent research has suggested that different nuDNA backgrounds can accommodate mutations occurring in mtDNA (e.g., [78], [79]). Thus, cytonuclear discordance might not be a problem. Further, if eco-climate drives selection on the mt genome, then strong selection would likely occur in some of the hundreds of nuDNA genes involved in OXPHOS. Such simultaneous selection on multiple genes should result in the speciation of differing matrilines owing to selection against hybrid offspring; the offspring would have inefficient or nonfunctional OXPHOS compared to pure coevolved lineages. Where tested with nuDNA genes, gene flow appears to be unabated across the mtDNA discontinuities [3], [6], [47], [48], [52]. Thus, nuDNA evidence does not support the eco-climate scenario.

In addition to the midpeninsular mtDNA discordance, differences in ecology, climate and volcanism fail to account for the other peninsular mtDNA discontinuities, including those near Loreto, the Isthmus of La Paz and in the southern Cape Region. Consequently, ephemeral seaways, such as the one at the Isthmus of La Paz and the theoretical Vizcaíno Seaway [5], provide the only viable explanation for the origin of coincidental mtDNA discordances. All other explanations await sound evidence.

Based on the matrilineal genealogy, statistical parsimony network and Bayesian skyline plot, the origin of *C. spinatus* appears to be in the southern part of the peninsula and on at least one island. The population on Isla Carmen appears to be a relic of past distributions and antiquity is likely for the Cape Region because it contains the greatest density of haplotypes and a great number of mutational steps separate it from other areas. The northward expansion from these areas, and the absence of a midpeninsular discontinuity, suggests a recent occurrence. The negative values of Fu's *Fs* and Tajima's *D*, and the Bayesian skyline plot support this scenario. When? During the LGM, *C. spinatus* may have been restricted to the southern peninsula. When the climate began to warm and desertification increased, *C. spinatus* dispersed northwards.

*Chaetodipus spinatus* occurs on islands within 15 km of the peninsula, but not on those farther away. Further, the species does not co-inhabit islands with other species of rodents, including other species of *Chaetodipus*. The absence of sympatry hints at competitive exclusion, given the ability of *C. spinatus* to dwell on islands. This distribution may reflect an expansion concomitant with the increase in global temperature and the formation of landbridge islands due to glacial melt. Fig. 5 shows that increases in the mean sea-level [60] lag about 2,000 yr behind increases of sea-surface temperatures [12] and this corresponds to implied increases in the population size of *C. spinatus*. The delay between the increase in the temperature and the expansion of *C. spinatus* may relate directly to the change in vegetation from mesic pine-oak forests to the current desert flora. Under mesic conditions, *Peromyscus*, mainly of the *P. maniculatus* group, could be the most common species. These mice persist today in mesic areas, mainly on the cool and wet Pacific side of the peninsula.

The historical origins of mice on most islands reflect Pleistocene sea-level changes. During the northward expansion of *C. spinatus* from the Cape Region beginning about 7.5 Ka, rapidly rising sea-level would have closed most landbridges. Islands would have trapped more species with mesic affiliations (Cricetidae) and less desert-adapted species (Heteromyidae). Heteromyids, especially species of *Chaetodipus*, would dominate the islands. Thus, it is not surprising that *C. spinatus* is the most wide-ranging mouse on the peninsula, and abundant mammal on the islands.

## Appendix 1(Haplotypes of Cytochrome *b*)

*California:* Tumco (**A**) Haplotypeype 1 (KM980312), California (32.8871–114.8280; 195185 MVZ).

*Baja California:* San Felipe (**B**) Haplotype 2 (KM980313), 1 km W San Felipe (31.0227–114.8416; 3125 CIB); haplotype 3 (KM980314), 1 km W San Felipe (31.0227–114.8416; 3132 CIB); haplotype 4 (KM980315), 1 km W Mission San Fernando (29.9666–115.25; 3150 CIB). Gonzaga Bay (**C**) Haplotype 5 (KM980316), 23 km N, 21 km W Bahía San Luis Gonzaga (29.9575–114.5025; 3086; 3088 CIB); haplotype 6 (KM980317), 23 km N, 21 km W Bahía San Luis Gonzaga (29.9575–114.5025; 3087 CIB); haplotype 7 (KM980318), 23 km N, 21 km W Bahía San Luis Gonzaga (29.9575–114.5025; 3089 CIB); haplotype 8 (KM980319), 23 km N, 21 km W Bahía San Luis Gonzaga (29.9575–114.5025; 3091 CIB). Gonzaga Island (**D**) Haplotype 9 (KM980320), Isla San Luis Gonzaga (29.8172–114.4041; 1045; 1050, 1051, 1052, 1046 CIB). Bahia de los Angeles (**E**) Haplotype 10 (KM980321), Smith, Bahia de los Angeles (28.9716–113.6061; 942 CIB); haplotype 11 (KM980322), 3 km N, 3 km W Bahia de Los Angeles (28.9716–113.6061; 7037 CIB); Tres Pachitas, 45 km S, 12 km E La Paz (23.74–110.2005; 7073 CIB); haplotype 12 (KM980323), 3 km N, 3 km W Bahía de Los Angeles (28.9716–113.6061; 7038 CIB). Las Animas Island (**F**) Haplotype 13 (KM980324), Isla Animas Island (28.7–112.9347; 1105, 1106, 1109, 1110, 1120 CIB); haplotype 13 (KM980324), Isla San Lorenzo Norte, near E (28.7617–112.9327; 1111; 1117 CIB). San Lorenzo Island (**G**) Haplotype 5 (KM980316), Isla San Lorenzo Sur, near NW (28.6666–112.875; 1167 CIB); haplotype 14 (KM980325), Isla San Lorenzo Sur, near NW (28.6666–112.875; 1146; 1147; 1149; 1153; 1154; 1155; 1156; 1158).

*Baja California Sur*: Vizcaino (**H**) Haplotype 3 (KM980314), 1 km S 1 km W, San Francisco de la Sierra (27.5636–113.0763; 8782 CIB); "El Monte" 45 km N, 31.5 km E Guerrero Negro (28.3488–113.6863; 10328 CIB); haplotype 9 (KM980320), "El Monte" 45 km N, 31.5 km E Guerrero Negro (28.3488–113.6863; 10326 CIB); 33 km N 5 km W, San Ignacio (27.5436–112.9680; 8776 CIB); haplotype 15 (KM980326), "El Monte" 45 km N, 31.5 km E Guerrero Negro (28.3488–113.6863; 10325 CIB); haplotype 16 (KM980327), "El Monte" 45 km N, 31.5 km E Guerrero Negro (28.3488–113.6863; 10327 CIB); haplotype 18 (KM980329), San Francisco de la Sierra (27.5899–113.0922; 3118 CIB); haplotype 19 (KM980310), 1 km S 1 km W, San Francisco de la Sierra (27.5636–113.0763; 8783 CIB); haplotype 20 (KM980330), 33 km N, 5 km W San Ignacio (27.5436–112.9680; 8773 CIB); haplotype 21 (KM980331), 33 km N 5 km W, San Ignacio (27.5436–112.9680; 8775 CIB); haplotype 22 (KM980332), 33 km N 5 km W, San Ignacio (27.5436–112.9680; 8779 CIB); haplotype 23 (KM980333), 33 km N 5 km W, San Ignacio (27.5436–112.9680; 8781 CIB). Santa Rosalia (**I**) Haplotype 3 (KM980314), 10 km N, 14 km W, Santa Rosalia (27.4044–112.4260; 2530 CIB); haplotype 9 (KM980320), 7.3 km N, 8.5 km W Santa Rosalia (27.3855–112.3622; 10305, 10308 CIB); haplotype 24 (KM980334), 10 km N, 14 km W, Santa Rosalia (27.4044–112.4260; 2529 CIB); haplotype 25 (KM980335), 7.3 km N, 8.5 km W Santa Rosalia (27.3855–112.3622; 10304 CIB); haplotype 26 (KM980336), 7.3 km N, 8.5 km W Santa Rosalia (27.3855–112.3622; 10299 CIB); haplotype 27 (KM980309), 7.3 km N, 8.5 km W Sta. Rosalia (27.3855–112.3622; 10301 CIB); haplotype 44 (KM980353), 1.5 km S, 1.1 km E San Bruno (26.2064–111.3989; 20008 CIB); haplotype 45 (KM980354), 1.5 km S, 1.1 km E San Bruno (26.2064–111.3989; 20009 CIB); haplotype 46 (KM980355), 1.5 km S, 1.1 km E San Bruno (26.2064–111.3989; 20007; 20011 CIB); haplotype 47 (KM980356), 1.5 km S, 1.1 km E San Bruno (26.2064–111.3989; 20010 CIB). San Marcos Island (J) Haplotype 28 (KM980337), Isla San Marcos (27.2444–112.0875; 380, 386; 387; 392 CIB). Punta Abreojos (K) Haplotype 29 (KM980338), 27 km N, 3 km E Abreojos (26.9788–113.4694; 8784 CIB); haplotype 30 (KM980339), 27 km N, 3 km E Abreojos (26.9788–113.4694; 8787 CIB); haplotype 31 (KM980340), 27 km N, 3 km E Abreojos (26.9788–113.4694; 8788 CIB); haplotype 32 (KM980341), 27 km N, 3 km E Abreojos (26.9788–113.4694; 8786 CIB). San Juanico (L) Haplotype 17 (KM980328), "El Monte" 45 km N, 31.5 km E Guerrero Negro (28.3488–113.6863; 10324 CIB); L Haplotype 36 (KM980345), "El Internado", San Miguel de Comondu (26.0708–111.8423; 5677 CIB); L Haplotype 38 (KM980347), 40 km N, 5 km E San Juanico (26.4827–112.6872; 9516 CIB); haplotype 39 (KM980348), 40 km N, 5 km E San Juanico (26.4827–112.6872; 9515 CIB); haplotype 40 (KM980349), 18.1 km N 24 km E, La Purísima (26.3591–111.8525; 9969 CIB); haplotype 41 (KM980350), 18.1 km N 24 km E, La Purisima (26.3591–111.8525; 9971 CIB); haplotype 42 (KM980351), 6.7 km N, 8.9 km E La Purisima (26.2536–112.0038; 10317 CIB); haplotype 43 (KM980352), 6.7 km N, 8.9 km E La Purisima (26.2536–112.0038; 10319 CIB); haplotype 47 (KM980356), 15.7 km N 21.6 km E Cd. Insurgentes (25.3969–111.5644; 9979 CIB); haplotype 65 (KM980372), 15.7 km N 21.6 km E Cd. Insurgentes (25.3969–111.5644; 9985 CIB); haplotype 66 (KM980373), 15.7 km N, 21.6 km E Ciudad Insurgentes (25.3969–111.5644; 9984 CIB); haplotype 119 (KM980424), San Vicente de la Sierra, 19.7 km S, 14.4 km E Pescadero (23.1997–109.9844; 1185 CIB); M Haplotype 9 (KM980320), San Pedro de la Sierra 6 km S, 4.8 km W Mulege (26.8997–112.5163; 8225 CIB). Mulege (M) Haplotype 33 (KM980342), San Pedro de la Sierra 6 km S, 4.8 km W Mulege (26.8997–112.5163; 8216 CIB); haplotype 34 (KM980343), San Pedro de la Sierra 6 km S, 4.8 km W Mulege (26.8997–112.5163; 8215 CIB); haplotype 35 (KM980344), San Pedro de la Sierra 6 km S, 4.8 km W Mulege (26.8997–112.5163; 8218 CIB); haplotype 36 (KM980345), San Pedro de la Sierra 6 km S, 4.8 km W Mulege (26.8997–112.5163; 8217 CIB); haplotype 37 (KM980346), San Pedro de la Sierra 6 km S, 4.8 km W Mulege (26.8997–112.5163; 8219; 8221 CIB). Coronados Island (N) Haplotype 51 (KM980358), Isla Coronados (26.1144–111.2802; 5851; 5856 CIB); haplotype 52 (KM980359), Isla Coronados (26.1144–111.2802; 5849, 5850, 5853 CIB). Loreto (O) Haplotype 40 (KM980349), 4 km S Loreto (25.9988–111.3436; 6387 CIB); haplotype 50 (KM980357), Rancho San Pedro, 35 km NW Loreto (26.1983–111.5758; 5676 CIB); haplotype 53 (KM980360), 4 km S Loreto (25.9988–111.3436; 6385 CIB); haplotype 54 (KM980361), 6.7 km S, 1.3 km W Loreto (25.9399–111.3618; 11125 CIB); haplotype 55 (KM980362), 6.7 km S, 1.3 km W Loreto (25.9399–111.3618; 11127 CIB); haplotype 56 (KM980363), 8.2 km S, 781 m W Loreto (25.9247–111.3566; 11130 CIB); haplotype 64 (KM980371), 28.8 km S, 9.3 km E Loreto (25.7389–111.2550; 11138 CIB). Isla Carmen (P) Haplotype 57 (KM980364), Isla Carmen (25.8305–115.2347; 407 CIB); haplotype 58 (KM980365), Isla Carmen (25.8305–115.2347; 410 CIB); haplotype 59 (KM980366), Isla Carmen (25.8305–115.2347; 409, 411 CIB); haplotype 60 (KM980367), Isla Carmen, Playa Márquez (25.8305–115.2347; 5706 CIB); haplotype 61 (KM980368), Isla Carmen (25.8305–115.2347; 412 CIB). Danzante Island (Q)Haplotype 62 (KM980369), Isla Danzante (25.7666–111.2527; 456, 457, 462, 466, 468 CIB); haplotype 63 (KM980370), Isla Danzante (25.7666–111.2527; 463 CIB). San José Island (R)Haplotype 67 (KM980374), Isla San Jose (24.95–110.6513; 347 CIB); Punta SW, Isla San Jose (24.9–110.5833; 1063, 1081 CIB); haplotype 68 (KM980375), Isla San José (24.9–110.5833; 348 CIB); haplotype 69 (KM980376), Isla San José, near SW cerca de la salina (24.9–110.5833; 1061 CIB); haplotype 70 (KM980377), Isla San José, near SW cerca de la salina (24.9–110.5833; 1062 CIB). San Pedro (S)Haplotype 66 (KM980373), San Pedro de la Presa 460 m (24.8708–111.0552; 6401 CIB); haplotype 71 (KM980378), San Evaristo 67 km N, San Juan de la Costa (24.8933–110.7172; 8801 CIB); S Haplotype 72 (KM980379), San Evaristo 67 km N, San Juan de la Costa (24.8933–110.7172; 8808 CIB); haplotype 73 (KM980380), San Evaristo 67 km N, San Juan de la Costa (24.8933–110.7172; 8800 CIB); haplotype 74 (KM980381), San Evaristo 67 km N, San Juan de la Costa (24.8933–110.7172; 8803 CIB); haplotype 75 (KM980382), San Pedro de la Presa 460 m (24.8708–111.0552; 6398 CIB); haplotype 76 (KM980383),San Pedro de la Presa 460 m (24.8708–111.0552; 6402 CIB); haplotype 77 (KM980384), San Pedro de la Presa 460 m (24.8708–111.0552; 6403 CIB); haplotype 78 (KM980385), San Pedro de la Presa 460 m (24.8708–111.0552; 6395 CIB); Pénjamo, 8 km N, 6 km W El Cién (24.4808–111.0838; 6412, 6413 CIB); haplotype 87 (KM980393), Pénjamo, 8 km N, 6 km W El Cien (24.4808–111.0838; 6411 CIB); haplotype 93 (KM980399), 18 km S, 9 km E El Cien (24.2455–111.0334; 6414 CIB). San Francisco Island (T) Haplotype 9 (KM980320), Islas San Francisco (24.8347–110.5722; 364, 366–369 CIB); haplotype 79 (KM980386), Isla San Francisco (24.8347–110.5722; 371 CIB); haplotype 80 (KM980387), Islas San Francisco (24.8347–110.5722; 365 CIB). Magdalena Island (U) Haplotype 81 (KM980388), Isla Magdalena, Punta Arena (24.6410–112.1571; 5096 CIB); haplotype 82 (KM980389), Isla Magdalena, Punta Arena (24.6410–112.1571; 5094, 5095, 5097, 5099 CIB). Margarita Island (V) Haplotype 18 (KM980329), 3 km SW Puerto Cortés, Isla Margarita (24.4486–111.8413; 6016 CIB); Isla Margarita (24.509–111.8568; 5110 CIB); haplotype 83 (KM980390), Isla Margarita (24.509–111.8568; 5105 CIB); Isla Margarita, 3 km NW Puerto Cortes (24.509–111.8568; 5987 CIB); Isla Margarita, 2 km N Puerto Alcatraz, (24.509–111.8568; 5111 CIB); 2 km N Puerto Alcatraz, Isla Margarita (24.509–111.8568; 5106 CIB); haplotype 85 (KM980391), Isla Margarita (24.509–111.8568; 5108 CIB); haplotype 86 (KM980392), Isla Margarita (24.509–111.8568; 6017 CIB); haplotype 92 (KM980398), 3 km SW Puerto Cortés, Isla Margarita (24.4486–111.8413; 6014 CIB). Espiritu Santo Island (W) Haplotype 88 (KM980394), Isla Espíritu Santo, El Candelero (24.45–110.3666; 4936 CIB); haplotype 89 (KM980395), Isla Espíritu Santo, El Candelero (24.45–110.3666; 358, 361 CIB); haplotype 90 (KM980396), Isla Espíritu Santo, El Candelero (24.45–110.3666; 360 CIB); haplotype 91 (KM980397), Isla Espíritu Santo, El Candelero (24.45–110.3666; 359 CIB). La Paz (X) Haplotype 94 (KM980400), "El Comitán" 17.5 km W La Paz (24.1375–110.4673; 2187 CIB); haplotype 95 (KM980401), "El Comitán" 17.5 km W La Paz (24.1375–110.4673; 2195 CIB); haplotype 96 (KM980402), "El Comitán" 17.5 km W La Paz (24.1375–110.4673; 2188, 2189 CIB); haplotype 97 (KM980403), "El Comitán" 17.5 km W La Paz (24.1375–110.4673; 2190 CIB); haplotype 101 (KM980407), 7 km S, 31 km W La Paz (24.0828–110.6012; 6425 CIB). Los Planes (Y) Haplotype 98 (KM980404), 16.1 km N, 6.4 km W Los Planes (24.1121–110; 19517, 19519 CIB); haplotype 99 (KM980405), 16.1 km N, 6.4 km W Los Planes (24.1121–110; 19516 CIB); haplotype 100 (KM980406), 16.1 km N, 6.4 km W Los Planes (24.1121–110; 19518 CIB); haplotype 102 (KM980408), 12.4 km N, 17.7 km W Los Planes (24.0794–110.1098; 19521 CIB); haplotype 103 (KM980409), 12.4 km N, 17.7 km W Los Planes (24.0794–110.1098; 19520 CIB); haplotype 104 (KM980410), 10 km N, 10 km W Los Planes (24.0612–110.0345; 18997 CIB); haplotype 105 (KM980411), 10 km N, 10 km W Los Planes (24.0612–110.0345; 18994 CIB); haplotype 106 (KM980412), 10 km N, 10 km W Los Planes (24.0612–110.0345; 18996 CIB). Cape Region (Z) Haplotype 9 (KM980320), 3 km N, 0.350 km E Los Barriles (23.6331–109.6753; 11166; 11164 CIB); haplotype 52 (KM980359), Rancho " La Misión", Santiago (23.4613–109.7231; 5847 CIB); haplotype 107 (KM980413), Tres Pachitas, 45 km S, 12 km E La Paz (23.74–110.2005; 7048, 7053 CIB); haplotype 108 (), Tres Pachitas, 45 km S, 12 km E La Paz (23.74–110.2005; 7058 CIB); haplotype 108 (KM980414), Tres Pachitas, 45 km S, 12 km E La Paz (23.74–110.2005; 7051 CIB); haplotype 109 (KM980415), Tres Pachitas, 45 km S, 12 km E La Paz (23.74–110.2005; 7049 CIB); haplotype 11 (KM980322), Tres Pachitas, 45 km S, 12 km E La Paz (23.74–110.2005; 7073 CIB); haplotype 110 (KM980416), San Bartolo (23.6978–109.799; 427 CIB); haplotype 111 (KM980311), 1.6 km S, 3 km E Los Barriles (23.6214–109.6492; 11160 CIB); haplotype 112 (KM980417), El Vergel, 12 km NW Santiago (23.5102–109.8358; 8211 CIB); haplotype 113 (KM980418), El Vergel, 12 km NW Santiago (23.5102–109.8358; 8213 CIB); haplotype 114 (KM980419), El Vergel, 12 km NW Santiago (23.5102–109.8358; 8205 CIB); haplotype 115 (KM980420), El Vergel, 12 km NW Santiago (23.5102–109.8358; 8207 CIB); haplotype 116 (KM980421), El Vergel, 12 km NW Santiago (23.5102–109.8358; 8214 CIB); haplotype 117 (KM980422), 3 km S Pescadero (23.3525–110.1891; 6437 CIB); haplotype 118 (KM980423), San Vicente de la Sierra, 19.7 km S, 14.4 km E Pescadero (23.1997–109.9844; 11759 México); haplotype 120 (KM980425), Santa Anita (23.1822–109.7186; 6442 CIB); haplotype 121 (KM980426), Santa Anita (23.1822–109.7186; 6445 CIB); haplotype 122 (KM980427), Santa Anita (23.1822–109.7186; 6451 CIB); haplotype 123 (KM980428), Santa Anita (23.1822–109.7186; 6443 CIB); haplotype 124 (KM980429), Santa Anita (23.1822–109.7186; 6446 CIB); Z Haplotype 125 (KM980430), San Vicente de la Sierra, 19.7 km S, 14.4 km E Pescadero (23.1729–109.9850; 11643 México); haplotype 126 (KM980431), San Vicente de la Sierra, 19.7 km S, 14.4 km E Pescadero (23.1729–109.9850; 11642 México); haplotype 127 (KM980432), San José del Cabo (23.0608–109.6863; 6472, 6475 CIB); haplotype 128 (KM980433), San Vicente de la Sierra, 19.7 km S, 14.4 km E Pescadero (23.1997–109.9844; 11691 CIB); haplotype 129 (KM980434), San Vicente de la Sierra, 19.7 km S, 14.4 km E Pescadero (23.1997–109.9844; 11692 CIB); haplotype 130 (KM980435), 6 km SE Migriño (23.0222–110.0683; 6469 CIB); haplotype 131 (KM980436), 6 km SE Migriño (23.0222–110.0683; 6470 CIB); haplotype 132 (KM980437), 6 km SE Migriño (23.0222–110.0683; 6467 CIB); haplotype 133 (KM980438), 6 km SE Migriño (23.0222–110.0683; 6468 CIB).

## Appendix 2(Haplotypes of Cytochrome Oxidase Subunit One)

Haplotype 1 (KM980203), San Felipe, 1 km W San Felipe (31.0228–114.8420; 3125 CIB). Haplotype 2 (KM980204), 23 km N, 21 km W Bahia San Luis Gonzaga (29.9540––114.5070; 3087 CIB). Haplotype 3 (KM980205), 3 km N, 3 km W Bahia de Los Angeles (28.9717–113.6060; 7037 CIB); San Pedro de la Sierra, 6 km S, 4.8 km W Mulege (26.8997–112.5159; 8215 CIB); San Zacarias, 13 km S, 3 km E San Ignacio (27.1420–112.9130; 9513 CIB). Haplotype 4 (KM980206), Isla San Lorenzo, Isla San Lorenzo Norte, near E (28.7619––112.9329; 1108, 1112–1116, 1120 CIB). Haplotype 5 (KM980207), Isla San Lorenzo Norte, near E. (Isla Las Animas) (28.7000–112.9300; 1109 CIB). Haplotype 6 (KM980208), Isla San Lorenzo, Isla San Lorenzo Sur, near NW (28.6667–112.875; 1146 CIB). Haplotype 7 (KM980209), El Monte, 45 km N, 31.5 km E Guerrero Negro (28.3488–113.6859; 10324 CIB). Haplotype 8 (KM980210), 33 km N 5 km W, San Ignacio (27.5436–112.9680; 8775 CIB). Haplotype 9 (KM980211), 27 km N, 3 km E Abreojos (26.9789–113.4690; 8786 CIB). Haplotype 9 (KM980211), San Ignacio (27.2989–112.8939; 11989 CIB). Haplotype 10 (KM980212), Isla San Marcos (27.2439–112.0879; 379 CIB); Isla San Marcos (27.2444–112.0875; 379, 380, 382, 383, 384, 392 CIB). Haplotype 11 (KM980213), Isla San Marcos (27.2444–112.0875; 381 CIB). Haplotype 12 (KM980214), Isla San Marcos (27.2439–112.0879; 385 CIB). Haplotype 13 (KM980215), Isla San Marcos (27.2439–112.0879; 391 CIB). Haplotype 14 (KM980216), Isla San Marcos (27.2439–112.0879; 393 CIB). Haplotype 15 (KM980217), 6.4 Km S, 2.8 Km E San Ignacio (27.2220–112.8720; 1450 CIB). Haplotype 16 (KM980218), 6.4 Km S, 2.8 Km E San Ignacio (27.2220–112.8720; 1451 CIB). Haplotype 17 (KM980219), 6.4 Km S, 2.8 Km E San Ignacio (27.2220–112.8720; 1452 CIB). Haplotype 18 (KM980220), 6.4 Km S, 2.8 Km E San Ignacio (27.2220–112.8720; 1453 CIB). Haplotype 19 (KM980221), 6.4 Km S, 2.8 Km E San Ignacio (27.2220–112.8720; 1454 CIB). Haplotype 20 (KM980222), 6.4 Km S, 2.8 Km E San Ignacio (27.2220–112.8720; 1455 CIB). Haplotype 21 (KM980223), 6.4 Km S, 2.8 Km E San Ignacio (27.2220–112.8720; 1456 CIB). Haplotype 22 (KM980224), 40 km N, 5 km E San Juanico (26.4827–112.6869; 9516 CIB). Haplotype 23 (KM980225), 17.2 km N, 7.5 km W Loreto (26.1613–111.4339; 17800 CIB). Haplotype 24 (KM980226), 17.2 km N, 7.5 km W Loreto (26.1613–111.4339; 17801 CIB). Haplotype 25 (KM980227), 17.2 km N, 7.5 km W Loreto (26.1613–111.4339; 17802 CIB). Haplotype 26 (KM980228), Playa Arenosa, Isla Coronados (26.1144–111.2802; 5851 CIB). Haplotype 27 (KM980229), Playa Arenosa, Isla Coronados (26.1144–111.2802; 5853 CIB). Haplotype 28 (KM980230), Playa Arenosa, Isla Coronados (26.1140–111.2799; 5849 CIB). Haplotype 28 (), Playa Arenosa, Isla Coronados (26.1140–111.2799; 5856 CIB). Haplotype 29 (KM980231), Isla Carmen (26.0240–111.1640; 406, 408, 410, 412 CIB). Haplotype 30 (KM980232), Isla Carmen (26.0240–111.1640; 405, 409, 411 CIB). Haplotype 31 (KM980233), 6.7 km S, 1.3 km W Loreto (25.9398–111.3619; 11125 CIB). Haplotype 32 (KM980234), Playa Marquez, Isla Carmen (25.8640–111.2200; 5706 CIB). Haplotype 33 (KM980235), Isla Carmen (25.8148–111.2089; 407 CIB). Haplotype 34 (KM980236), Isla Danzante (25.7682–111.2459; 445–453, 456, 457, 463 CIB). Haplotype 35 (KM980237), 15.7 km N, 21.6 km E Ciudad Insurgentes (25.3969–111.5640; 9979 CIB). Haplotype 36 (KM980239), Isla San Jose Parte NE (25–110.5830; 11005, 11008 CIB). Haplotype 37 (KM980240), Isla San Jose Parte NE (25–110.5830; 11006 CIB). Haplotype 38 (KM980241), Isla San Jose Parte NE (25–110.5830; 11009 CIB). Haplotype 39 (KM980242), Isla San Jose Parte NE (25–110.5830; 11010 CIB). Haplotype 40 (KM980243), Isla San Jose (24.9500–110.6510; 13393 CIB). Haplotype 41 (KM980244), Isla San Jose (24.9500–110.6510; 1081, 13395, 13397 CIB). Haplotype 42 (KM980245), Isla San Jose (24.95–110.6513; 13394 CIB). Haplotype 43 (KM980246), Isla San Jose (24.95–110.6513; 13396 CIB). Haplotype 44 (KM980247), San Evaristo, 67 km N San Juan de la Costa (24.8932–110.7170; 8801 CIB). Haplotype 45 (KM980248), Isla San Francisco. (San Francisquito) (24.8349–110.5719; 363–371, 376 CIB). Haplotype 46 (KM980249), 2 km N Puerto Alcatraz, Isla Margarita (24.5090–111.8570; 5105 CIB); 3 km SW Puerto Cortes, Isla Margarita (24.4489–111.8410; 6015 CIB). Haplotype 47 (KM980250), 2 km N Puerto Alcatraz, Isla Margarita (24.5090–111.8570; 5106, 5108, 5109 CIB); 3 km SW Puerto Cortes, Isla Margarita (24.4489–111.8410; 6014, 6017, 6041 CIB). Haplotype 48 (KM980251), 2 km N Puerto Alcatraz, Isla Margarita (24.5090–111.8570; 5107 CIB). Haplotype 49 (KM980252), Isla Espiritu Santo (24.4500–110.3669; 358. 362 CIB). Haplotype 50 (KM980253), Isla Espiritu Santo (24.4500–110.3669; 360, 361 CIB). Haplotype 51 (KM980254), Isla Espíritu Santo, El Candelero (24.45–110.3666; 359 CIB). Haplotype 52 (KM980255), 3 km SW Puerto Cortes, Isla Margarita (24.4489–111.8410; 6016 CIB). Haplotype 53 (KM980256), El Comitan, 17.5 km W La Paz (24.1375–110.4670; 2188 CIB). Haplotype 54 (KM980257), Tres Pachitas, 45 km S, 12 km E La Paz (23.7399–110.2009; 7049 CIB). Haplotype 55 (KM980258), Santa Anita (23.1821–109.7190; 6446 CIB). Haplotype 56 (KM980259), San Vicente de la Sierra, 19.7 km S, 14.4 km E Pescadero (23.1728–109.9850; 11643 CIB). Haplotype 57 (KM980260), San Jose del Cabo (23.0610–109.6859; 6472, 6475, 6478 CIB). Haplotype 58 (KM980261), San Jose del Cabo (23.0610–109.6859; 6476 CIB). Haplotype 59 (KM980262), San Jose del Cabo (23.0610–109.6859; 6477 CIB)
